# Two-Dimensional Materials for Raman Thermometry on Power Electronic Devices

**DOI:** 10.3390/nano15171344

**Published:** 2025-09-01

**Authors:** Mohammed Boussekri, Lucie Frogé, Raphael Sommet, Julie Cholet, Dominique Carisetti, Bruno Dlubak, Eva Desgué, Patrick Garabedian, Pierre Legagneux, Nicolas Sarazin, Mathieu Moreau, David Brunel, Pierre Seneor, Etienne Carré, Marie-Blandine Martin, Vincent Renaudin, Tony Moinet

**Affiliations:** 1 XLIM Laboratory, CNRS UMR 7252, University of Limoges, 19100 Brive, France; raphael.sommet@unilim.fr (R.S.); mathieu.moreau@unilim.fr (M.M.); 2 Thales Research and Technologies Palaiseau, 91120 Palaiseau, France; lucie.froge@thalesgroup.com (L.F.); julie.cholet@thalesgroup.com (J.C.); dominique.carisetti@thalesgroup.com (D.C.); eva.desgue@thalesgroup.com (E.D.); patrick.garabedian@thalesgroup.com (P.G.); pierre.legagneux@thalesgroup.com (P.L.); nicolas.sarazin@thalesgroup.com (N.S.); david.brunel@thalesgroup.com (D.B.); 3 Laboratoire Albert FERT Palaiseau, 91767 Palaiseau, France; bruno.dlubak@cnrs-thales.fr (B.D.); pierre.seneor@cnrs-thales.fr (P.S.); etienne.carre@onera.fr (E.C.); marie-blandine.martin@cnrs-thales.fr (M.-B.M.); 4 STMicroelectronics Tours & Grenoble, 38019 Grenoble, France; vincent.renaudin@st.com (V.R.); tony.moinet@st.com (T.M.)

**Keywords:** 2D materials, thermal sensors, Raman spectrometry, thermoreflectance, 3D finite elements simulations, power electronics

## Abstract

Raman thermometry is a powerful technique for sub-microscale thermal measurements on semiconductor-based devices, provided that the active region remains accessible and is not obscured by metallization. Since pure metals do not exhibit Raman scattering, traditional Raman thermometry becomes ineffective in such cases. To overcome this limitation, we propose the use of atomically thin Two-Dimensional materials as local temperature sensors. These materials generate Raman spectra at the nanoscale, enabling highly precise absolute surface temperature measurements. In this study, we investigate the feasibility and effectiveness of this approach by applying it to power devices, including a calibrated gold resistor and an SiC Junction Barrier Schottky (JBS) diode. We assess the processing challenges and measurement reliability of 2D materials for thermal characterization. To validate our findings, we complement Raman thermometry with thermoreflectance measurements, which are well suited for metallized surfaces. For example, on the serpentine resistor, Raman thermometry applied to the 2D material yielded a thermal resistance of 22.099 °C/W, while thermoreflectance on the metallic surface measured 21.898 °C/W. This close agreement suggests good thermal conductance at the metal/2D material interface. The results demonstrate the potential of integrating 2D materials as effective nanoscale temperature probes, offering new insights into thermal management strategies for advanced electronic components. Additionally, thermal simulations are conducted to further analyze the thermal response of these devices under operational conditions. Furthermore, we investigate two 2D material integration methods, transfer and direct growth, and evaluate them through measured thermal resistances for the SiC JBS diode, highlighting the influence of the deposition technique on thermal performance.

## 1. Introduction

**Figure 1:**
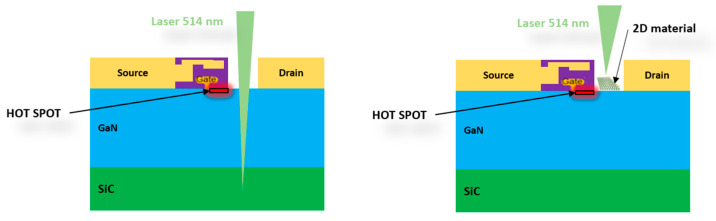
Schematic of junction temperature measurement in GaN-based HEMT devices using Raman spectroscopy: (**left**) without 2D material, (**right**) with a 2D material layer deposited above the gate region.

Power electronics based on wide bandgap semiconductors such as power SiC diodes, SiC MOSFETs, or GaN transistors are key components for high voltage, high switching frequency, and thermally constrained applications. This has led to significant market adoption in recent years for many applications, ranging from industrial rail traction inverters and high-efficiency devices for avionics to mainstream electric vehicles and energy conversion [1]. The lifetime and reliability of these high-power electronics are hence key issues in terms of product sustainability for the consumer and for the ecological necessity to produce durable products as well. To address this issue, the electro-thermal model of these power electronic devices should be as close as possible to the real component whose characteristics depend on packaging, environment, temperature gradient, and interfaces. Temperature measurement is one of the most accurate ways to (refine and/or) efficiently correct the model calculated by thermal simulation, which cannot take all parameters into account. Infrared (IR) thermography is now widely used for this kind of measurement and is well suited for thermal measurements on metallization at submicroscale. However, the IR technique is limited by its low resolution (depending on the detector wavelength range and Rayleigh criteria) and the low surface emissivity of materials commonly used in power devices such as aluminum (emissivity around 0.1). This problem can be overcome by applying a high-emissivity surface coating. Nevertheless, the coating generally affects the thermal resistance and the electrical performance of the device, preventing efficient measurements. Under optimal conditions, the accuracy is close to 10% for a spatial resolution of 3 µm in the middle wavelength infrared range (MWIR) [2]. On the other hand, thermoreflectance is a highly sensitive optical technique, with its high spatial, temporal, and temperature resolution (0.29 µm, 50 ns, and 0.5 °C, respectively) [3] that enables precise relative thermal measurements of power devices without the need for emissivity corrections. This makes it a valuable alternative to IR thermography, particularly for materials with low emissivity. Thermoreflectance is actually more employed for temperature gradient and thermal conductivity measurements [4] and often limited to laboratory/academic applications. Another widely used technique for thermal characterization is Raman spectroscopy, which has already provided valuable insights into the junction temperature of GaN-based high electron mobility transistors (HEMTs) with active area accessible to the 514 nm laser (Figure 1 left) [5]. The temperature was extracted from the Raman shift of the GaN E_2_ peak after a calibration step.

The popularity of the aforementioned method is due to its high spatial resolution (below 1 µm), its very high temperature sensitivity (close to 1 °C), and its unique ability to measure an absolute temperature from the intensity ratio of the Stokes and anti-Stokes signals. Such method is usually applied in cases of crystalline layers with high-intensity Raman phonon modes [6]. However, for transparent semiconductors (depending on the wavelength of the laser), this method gives only an average temperature over a relatively large depth near the hot spot (Figure 1 left).

In addition, these aforementioned methods are neither applicable to metallic layers due to interactions with plasmons which hide the Raman signature, nor applicable to amorphous layers due to the broadening of the Raman peaks. These issues can be solved by the use of a nanometer-scale temperature sensor made of monolayer MoS_2_ transferred onto the dye surface [7], or by using PtSe_2_ multilayers grown or transferred onto the components, which is the approach we propose (Figure 1 right) [8]. The use of 2D materials as temperature sensors has gained increasing attention in the scientific community due to their high thermal sensitivity, mechanical flexibility, and atomic-scale thickness. For example, in the field of wearable electronics, 2D materials such as graphene, MXenes, and TMDCs have been extensively explored as flexible temperature-sensing platforms [9], demonstrating their potential beyond laboratory environments and into practical applications.

To further enhance temperature characterization and validate Raman-based measurements, we used thermoreflectance as a complementary and validation technique. Thermoreflectance is applicable to a broader range of materials, including metallic and amorphous layers. By combining Raman spectrometry with thermoreflectance, a complete thermal characterization is achieved, ensuring accurate temperature extraction across different material layers in the device.

**Figure 2:**
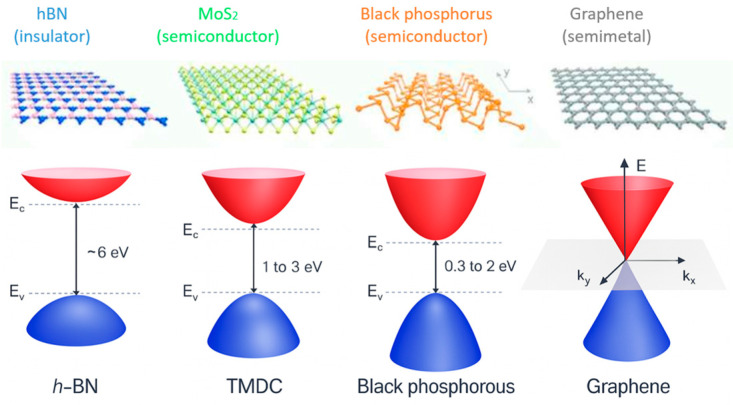
Energy spectrum and atomic crystal structures for monolayers of different two-dimensional (2D) materials. From left to right: boron nitride (h-BN), transition metal dichalcogenides (TMDCs), black phosphorous, and graphene. Adapted from [11].

Two-dimensional (2D) materials are intensively studied in different domains to exploit their intrinsic properties [10]. These crystalline materials are ultimately thin (one to a few atoms thick for one monolayer) and their Raman signatures allow precise measurement of the surface temperature. They can be transferred on top of surfaces ranging from wafer scale down to very small sub-µm^2^ areas (using 2D flakes) allowing from wide to ultra-localized thermal measurements. Alternatively, they can be synthesized directly on top of a die surface. The nature of the 2D material may be chosen among a wide list of insulators, semiconductors, and metals (Figure 2) to tailor their thermal and electrical properties according to the desired requirements and constraints of the devices.

In our project, we focus on 2D materials which give reliable results on 2D sensor manufacturing process and temperature extraction. Other labs also use nanoparticles of TiO_2_ or CeO_2_ in wet solution to do the same approach [12] but this could induce aggregates and non-uniformity of deposition by comparison with the deposition or transfer of 2D materials.

In this study, we transfer or directly synthesize PtSe_2_ patches on power devices to serve as nanoscale temperature probes. PtSe_2_ was chosen for its excellent chemical and thermal stability, high electrical conductivity, and air-stable high carrier mobility. Importantly, multilayer PtSe_2_ films grown by molecular beam epitaxy (MBE) have demonstrated remarkable long-term stability. After 1.5 years of exposure to ambient air, Raman spectra show no degradation of the Eg and A_1_g peak widths, the Se/Pt atomic ratio remains unchanged, and the sheet conductance exhibits only a minimal decrease—from 1.49 mS to 1.46 mS. These results confirm the robustness of PtSe_2_ in realistic operating environments, supporting its use as a reliable and durable material for thermal sensing in power electronics [13,14]. These 2D layers were grown by molecular beam epitaxy (MBE) either on sapphire substrate or directly on the active components. For the first process, the 2D layer is then transferred to the sample by using a polymer stamp of Polydimethylsiloxane (PDMS). In the second process, the 2D layer is directly grown on the whole surface of the active component and then locally patterned by projection photolithography and reactive ion etching (RIE). The proof of concept for surface thermal measurement is demonstrated on a serpentine gold resistor and on an SiC Junction Barrier Schottky (JBS) diode.

## 2. Two-Dimensional Patches Manufacturing

### 2.1. Two-Dimensional Materials Growth by MBE

Two-dimensional PtSe2 layers are grown in a 2-inch MBE reactor supplied by Dr Eberl MBE-Komponenten [14,15]. The selenium flux is supplied by a valved selenium cracker source and an electron beam evaporator generates the Pt flux from high purity (4N) Pt. For the synthesis of PtSe2 on c-plane sapphire substrate, the substrate is heated to 544 °C and simultaneously exposed to a Pt flux (0.003 Å/s) and a Se flux (0.2 Å/s). Concerning the direct synthesis on STMicroelectronics SiC diodes, the PtSe2 growth temperature is fixed at 400 °C and the fluxes are set to 0.003 and 0.5 Å/s for Pt and Se, respectively.

### 2.2. Two-Dimensional Transfer Technique from Initial Substrate to Power Devices

**Figure 3:**
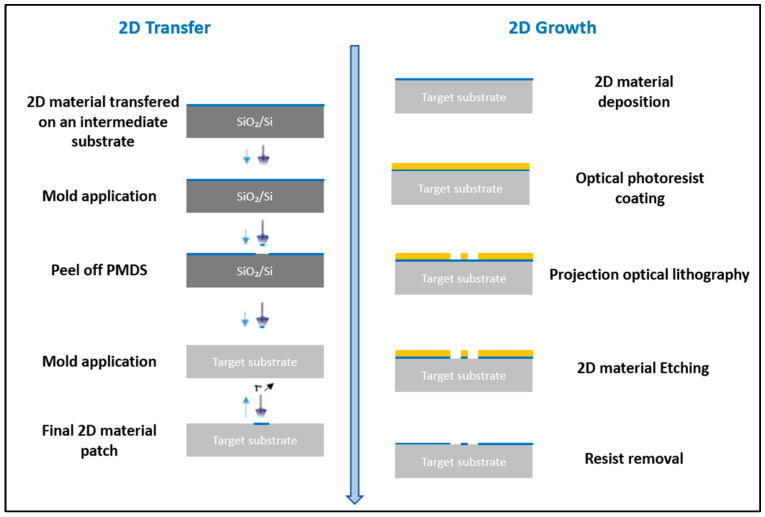
Experimental steps of the 2D material transfer (**left**) and the direct synthesis and growth on the target device (**right**).

**Figure 4:**
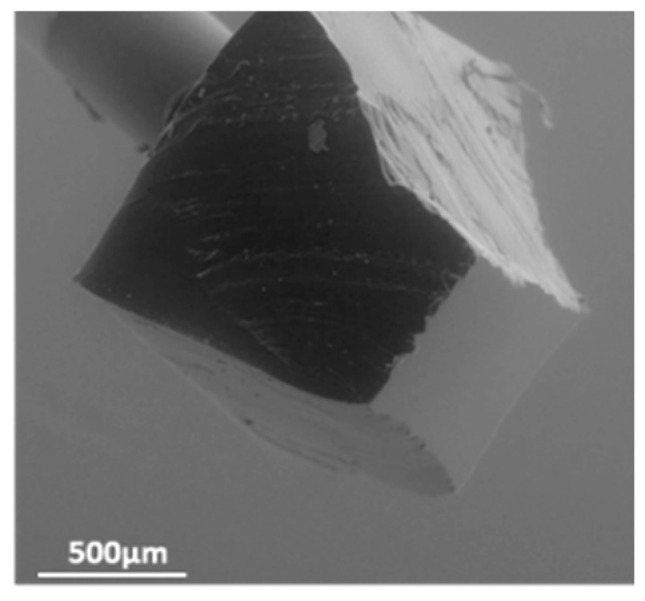
PDMS stamp with a surface contact area of approximatively 700 × 900 µm^2^.

**Figure 5:**
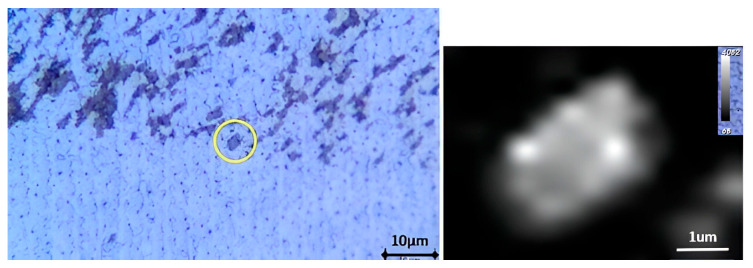
PtSe_2_ patch transferred on aluminum surface of an SiC diode (**left**) and Raman mapping of Eg peak intensity (**right**).

The use of a viscoelastic material such as Polydimethylsiloxane (PDMS) for the transfer of 2D materials is a clean conventional method [16]. In the case of the transfer on SiC diode, the roughness of the aluminum anode electrode at the chip surface and the local thickness difference at the edge of the chip makes it difficult for the application of the PDMS stamp. To overcome this challenge, we have developed a specific transfer process (Figure 3 left) relying on a PDMS tip made of Sylgard 184 attached to a needle, with a contact area (approximately 700 × 900 µm^2^) smaller than the chip surface (Figure 4). The whole structure is mounted on a setup to control the position of the tip and to modify the temperature in a specific chamber. The tip is first immersed in water to increase the adhesion energy between the PDMS and the 2D material. The PDMS stamp is then used to pick up the 2D material. The target substrate is placed in the temperature chamber for the transfer. The 2D flake is then released onto the target surface by applying the stamp to the surface and lowering the temperature, allowing the stamp to return to its initial shape and detach from the surface. Figure 5 shows the optical image of a PtSe_2_ patch successfully transferred to the top surface of an SiC diode as confirmed by the Raman mapping. Figure 3 (left) shows only the second stage of the 2D transfer process from the intermediate SiO_2_/Si substrate to the target substrate. However, this process involves an initial step where the 2D material is first transferred from its original sapphire growth substrate onto an intermediate SiO_2_/Si substrate using thermal release tape and a temporary gold support layer, which is subsequently etched.

### 2.3. Direct 2D Growth and Patches Manufacturing on Power Devices

**Figure 6:**
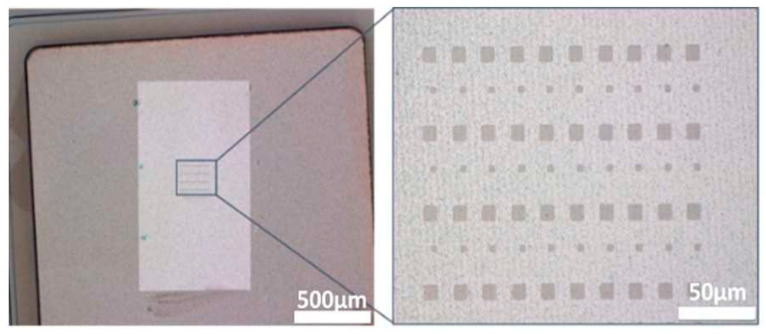
PtSe_2_ patches on an SiC diode using the “2D growth” method.

The second process investigated is to manufacture the 2D patch directly on the power device by direct synthesis and then patterning (Figure 3 right). A positive photoresist is deposited by spin coating on the target substrate covered with the 2D material. Projection optical lithography is used to pattern an array of photoresist patches of 5 × 5 µm^2^ and 10 × 10 µm^2^. Then, the unprotected 2D material is etched by RIE. Figure 6 shows PtSe_2_ patches realized on the aluminum anode electrode of an SiC diode.

The Raman signature of the 2D material is monitored at each step of the fabrication process for both aforementioned methods.

The E_g_ and A_1g_ Raman peaks (related to the in-plane and out-of-plane atomic vibrations, respectively) give information on the crystalline quality.

**Figure 7:**
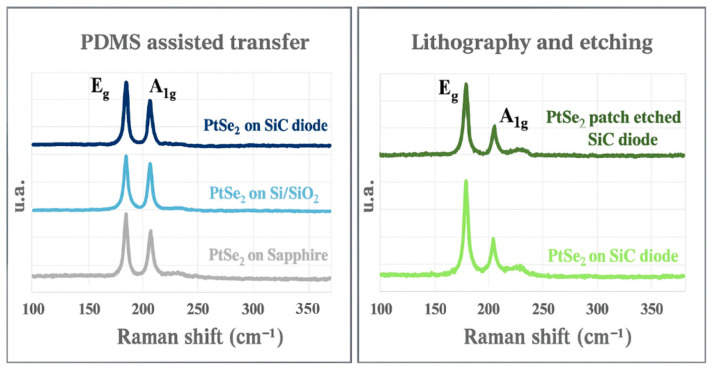
Raman signature during the PtSe_2_ patches manufacturing, using the transfer method (**left**) and the growth method (**right**).

As shown in Figure 7 (left), the transfer of the PtSe_2_ film (7.5 nm, 14 monolayers) from sapphire to SiO_2_/Si then to the SiC diode, is very satisfactory as the Raman signature of the PtSe_2_ film remains unchanged. For the final transfer to the sample, the A_1g_/E_g_ peak ratio is different but the crystalline quality is maintained [17]. For the direct growth method, no impact on the Raman signature is observed in Figure 7 (right), meaning that the PtSe_2_ layer is preserved after lithography and growth steps.

## 3. Experimental Setup for Thermal Measurements

### 3.1. Raman Spectroscopy Principle

The interaction of a monochromatic light (laser of frequency ω_0_) with the electrons of a material results in two effects:

(i) Elastic scattering with no change of light frequency (Rayleigh);
(ii) Inelastic scattering (Raman effect) involving atomic vibration modes (phonons) and change of frequency: ω_s_ = ω_0_ − ω_p_ for Stokes line (creation of a phonon) and ω_s_ = ω_0_ + ω_p_ for the anti-Stokes line (annihilation of a phonon). The Raman peaks (quasi-Lorentzian functions) are characteristic of the material investigated and depend on both temperature and stress. The Full Width Half Maximum (FWHM) of the peaks is very small allowing accurate monitoring of the peak shift and the intensity of the anti-Stokes and Stokes peaks. Moreover, due to its high spatial resolution (<1 μm), micro-Raman spectroscopy is a powerful tool for local measurements of temperature in micro-devices.

Two methods of Raman thermal measurements are investigated in this study:

(i) The temperature *T* relates to the intensity ratio *I_AS_*/*I_S_* of anti-Stokes and Stokes lines (AS/S), following the formula [18]:

(1) $$\frac{I_{AS}}{I_{S}}=Cexp(-\frac{\hbar \omega_{p}}{kT})$$

where ω_p_ is the frequency of a phonon mode and C, a calibration factor, ℏ the reduced Planck constant, *k* the Boltzmann constant, and *T* the temperature in Kelvin. This is a unique feature of Raman effect allowing the measurement of the absolute temperature of a material. However, the intensity of the anti-Stokes line must be strong enough which is satisfied for relatively low Raman frequency or high temperatures.

(ii) Temperature can also be measured from the Raman peak position. The temperature dependence of the Raman frequency ωp is approximately linear:

(2) $$\omega_{p}=\omega_{a}+S_{T}(T-T_{a})$$

where ω_a_, the Raman frequency measured at room temperature ($T_{a}$) depends both on the strain induced by the substrate and on the technology used. A calibration must be conducted on the same sample, prior to temperature measurements to determine $S_{T}$ (dω/dT), the sensitivity of Raman frequency for a variation of temperature. This factor depends on the material.

### 3.2. Instrumentation and Thermal Setup

**Figure 8:**
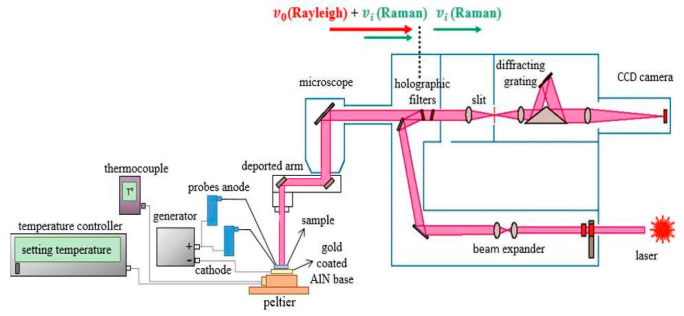
Schematic of thermal and electric setup for Raman temperature measurements.

A Renishaw Qontor µRaman spectrometer with a 514 nm 100 mW laser diode has been used for this study (Figure 8). For Stokes shift measurement (shift), a high-pass edge filter with 50 cm^−1^ cutoff is placed inside the optical path to access only the Stokes part of the spectrum for a better sensitivity. For anti-Stokes/Stokes (AS/S) peaks ratio, we used a reject band Notch filter to eliminate the Rayleigh response. The diffraction gratings exhibit 2400 lines/mm and 3000 lines/mm for a better accuracy, especially to facilitate the curve fitting. Peak position and intensity are extracted from fitting the Raman peaks with a quasi-Lorentzian function.

**Figure 9:**
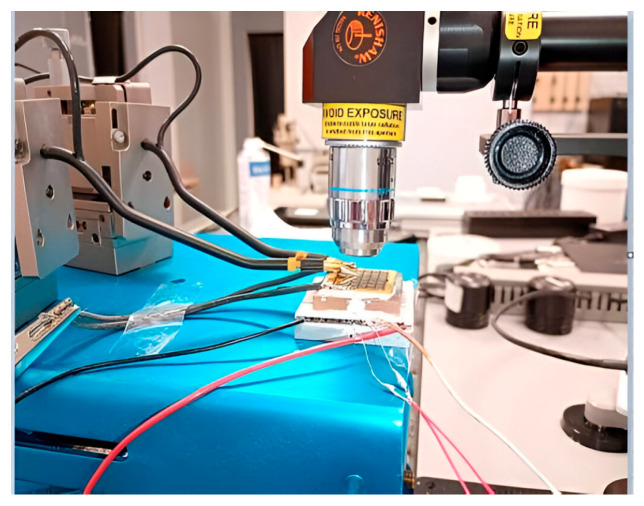
Optical view of the Raman measurements with electrical probes.

For thermal heating and device biasing, we used two kinds of setup. In all cases, the sample is placed on a Peltier thermoelectric (Figure 8 and Figure 9), which allows measurement from room temperature to 140 °C. As shown in Figure 9, the electrical DC bias of the device is performed directly by wires placed just below the microscope objective (X100 or X50).

### 3.3. Thermoreflectance Principle

The thermoreflectance technique is a non-contact, high-resolution method for measuring temperature variations on a device’s surface by analyzing changes in its optical reflectance. The sample (Device Under Test, DUT) reflects the incident light and the changes in reflectivity are detected by the CCD camera to extract temperature information with high spatial and temporal resolution. This method has proven to be a valuable tool for the thermometry of integrated circuits and semiconductor devices [19,20].

This technique is grounded in the following relationship:

(3) $$\frac{\Delta R}{R_{0}}=C_{th}\Delta T$$

**Figure 10:**
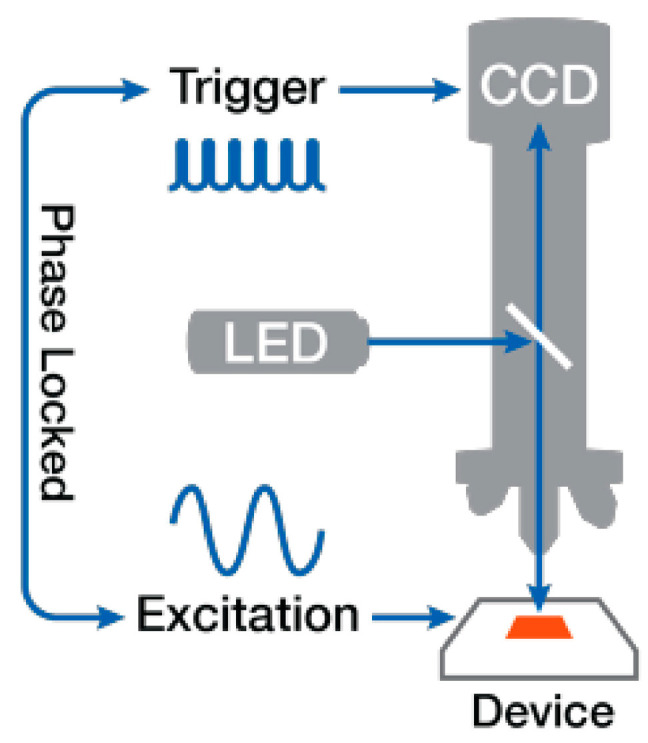
Schematic of the thermoreflectance setup.

**Figure 11:**
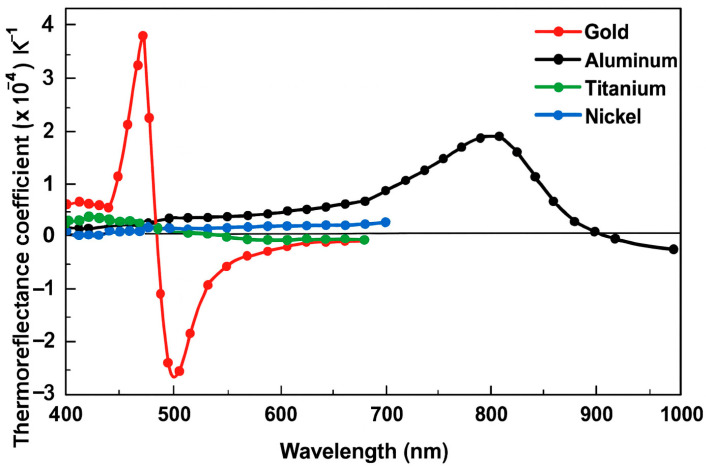
Variation of the thermal coefficient of reflectance $C_{th}$ as a function of wavelength for different materials.

The method begins with a calibration step, where a known temperature difference ΔT is applied to the device, usually using a thermal chuck, to determine the thermal coefficient of reflectance $C_{th}$ at specific wavelength λ. This coefficient depends not only on the material but also on the wavelength. In the measurement step, electrical power is dissipated through the device (Figure 10), causing a temperature rise. The system measures the change in reflectance and calculates the corresponding temperature using the pre-determined $C_{th}$(λ). As shown in Figure 11, $C_{th}$ varies with λ for different materials [21]. For gold, the optimum wavelengths are around 470 nm and 530 nm, while for aluminum, it is around 780 nm.

### 3.4. Thermoreflectance Thermal Setup

**Figure 12:**
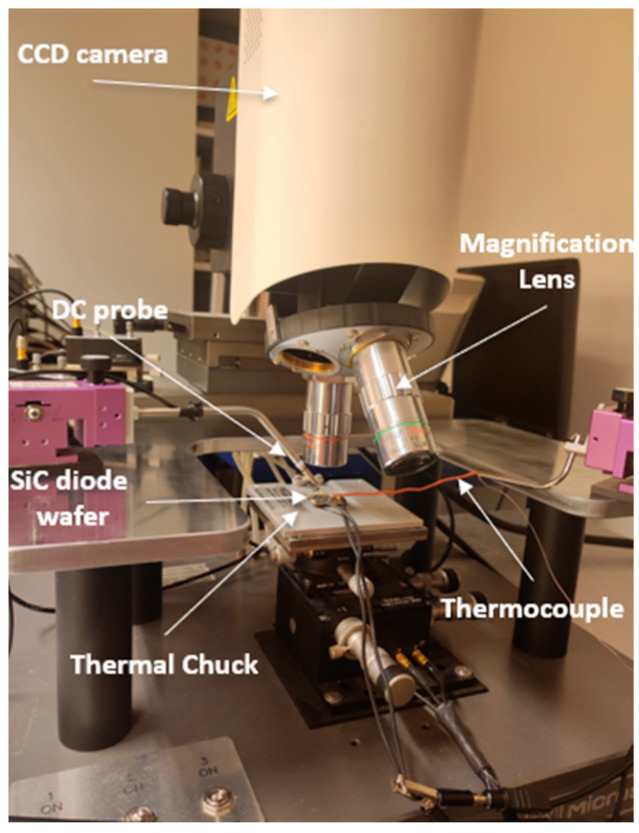
SiC diode wafer under test in thermoreflectance setup.

The thermoreflectance measurement setup is built around an NT220C setup from Microsanj (Figure 12). It consists of several key components to ensure precise thermal characterization of electronic devices. Inside the CCD camera, a light source with four different wavelengths (365 nm, 470 nm, 530 nm, and 780 nm) is used to illuminate the device. The LED excitation is synchronized with a phase-locked trigger to ensure accurate measurements (Figure 10). A range of magnification lenses (X5, X20, X50, and X100) allows for different spatial resolutions, enabling detailed thermal mapping at various scales. The heating of the DUT is controlled using a thermal chuck, which maintains a fixed ΔT during the calibration process. For gold surfaces, the estimated uncertainty of the thermoreflectance measurement is ±0.5 °C.

## 4. Experiments and Results

### 4.1. Serpentine Gold Resistors on Silicon Substrate

**Figure 13:**
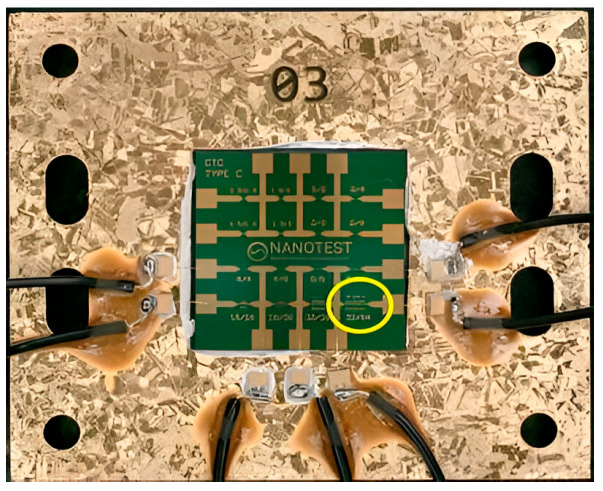
Optical image of the die with 16 resistors. The resistor used is highlighted in yellow and wires are connected to electrical setup.

To demonstrate the use of 2D materials as temperature sensors, we first validated the methodology on a gold resistor designed by Nanotest Inc. for thermal calibration (Figure 13).

**Figure 14:**
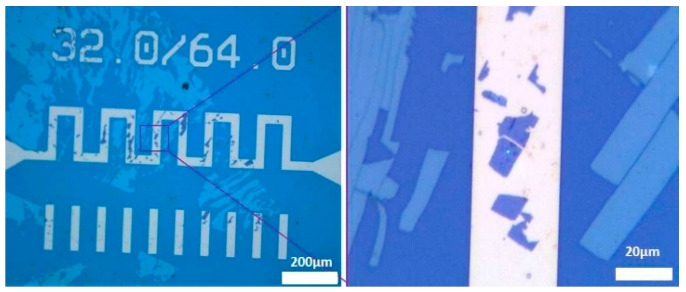
Optical images of the 32/64 resistor after the PtSe_2_ transfer by PDMS technique (**left**); zoom of the PtSe_2_ patch on the resistor with the laser spot for thermal Raman measurement (**right**).

The resistor is manufactured in Ti (10 nm)/Au (500 nm) deposited on SiO_2_/Si substrate. The structure is protected with a thin layer of 10 nm of Al_2_O_3_. The resistor analyzed is a serpentine of 1 mm long, 32 µm width, and 64 µm pitch (Figure 14 left). For electrical and thermal measurements, the die is glued with silver epoxy on a gold-coated Cu/Mo/Cu carrier and connected with gold wire bondings to metallized ceramic parts. Thick wires are brazed on the ceramics to connect the power supply.

#### 4.1.1. Raman Spectroscopy Results for the Gold Resistor

**Figure 15:**
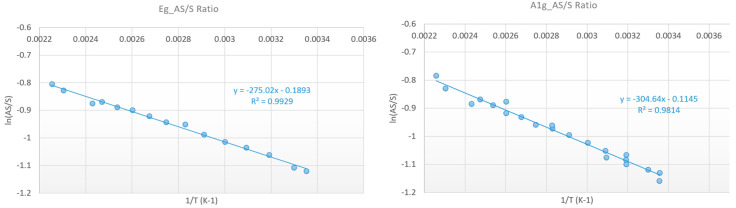
Thermal calibration curve with the AS/S Raman peak intensity ratio method. 50X, 2400 lines/mm, Notch filter, static regular, 0.25 mW, 30 s, 4 frames.

**Figure 16:**
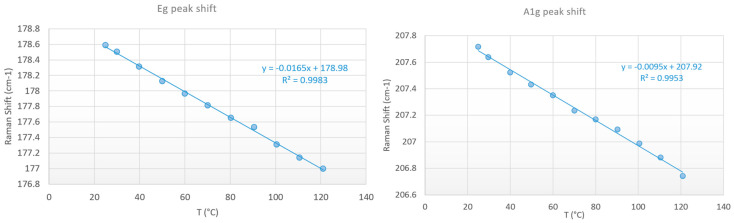
Thermal calibration curve with the Raman peak shift method. 50X, 3000 lines/mm, edge filter, static regular, 0.25 mW, 30 s, 2 frames.

MBE-grown PtSe_2_ layers were transferred on the resistor as described in Section 2.2. The Raman temperature measurement has been conducted on the PtSe_2_ patch which is very close to the middle of the resistor (see Figure 14), where we expected the hottest temperature. Calibration curves from room temperature to 120 °C with the use of the AS/S ratio (Equation (1)) are presented in Figure 15. The temperature is measured with a thermocouple fixed on the surface of the carrier. These measurements were carried out using a 50X objective, a 3000 lines/mm grating, an edge filter in static regular mode, with a laser power of 0.25 mW, an acquisition time of 30 s, and an accumulation of 2 frames. The laser power was adjusted in order to minimize the heating of the 2D patch and to optimize the results, especially for the anti-Stokes peak intensity. This leads to linear regressions with correlation coefficients R^2^ of 0.99 and 0.98 for the E_g_ and A_1g_ peak, respectively. Better results, with R^2^ ≥ 0.995, were found with calibration curves using the E_g_ and A_1g_ peak shift (Equation (2)) for the same temperature range (Figure 16).

The resistor is operated at different dissipated power and the gold-coated Cu/Mo/Cu carrier is maintained at T_case_ = 50 °C. This temperature is chosen for comparison with the measurements obtained using the IR technique. Temperatures corresponding to the E_g_ and A_1g_ peaks were extracted at various dissipated power levels ranging from 0 to 5.7 W.

Three different thermal measurement techniques were investigated: the shift of the Raman peaks, the Raman peak AS/S ratios, and IR measurements. Temperature values calculated from the shift and the AS/S of the E_g_ peak are very close, with a maximum temperature deviation of only 10 °C between the two methods at the highest tested temperature, and with R^2^ above 0.99 for both methods. In contrast, the use of the A_1g_ peak shift leads to much higher temperature deviation (up to +25 °C) compared to AS/S methods, demonstrating that the Eg peak shift gives more reliable results. The temperature difference between A_1g_ shift and AS/S methods is much too important, despite the good calibration curves. One of the causes could be the lower temperature dependence of the A_1g_ peak shift compared to the E_g_ peak shift (Figure 16 right) leading to higher uncertainties.

#### 4.1.2. Thermoreflectance Results for the Gold Resistor

**Figure 17:**
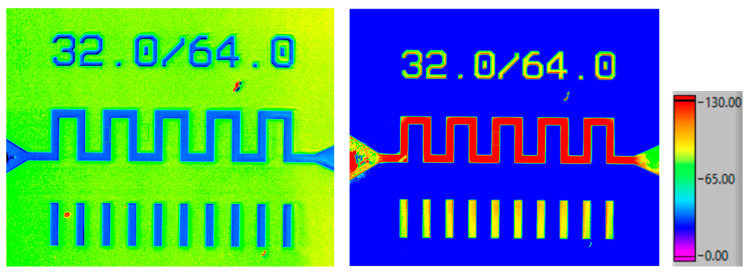
Thermoreflectance calibration image of the 32 × 64 resistor (**left**); thermoreflectance ΔT (°C) distribution of the 32 × 64 resistor for a dissipated power of 5.9 W (**right**).

The calibration of the thermoreflectance technique was conducted on a serpentine gold resistor model of 32 × 64 using a wavelength of λ = 530 nm. The blue regions in the thermoreflectance image (Figure 17 left) correspond to the gold material, which exhibits a thermoreflectance coefficient ($C_{th}$) of approximately −3.174 × 10^−4^ K^−1^. This value aligns closely with the experimentally measured models of $C_{th}$ as a function of wavelength (λ) for gold [22], confirming the consistency and reliability of the calibration process.

Following the calibration, we applied a range of dissipated power from 0.6 to 5.9 W to the resistor to investigate the thermal response. The initial temperature was approximately 28 °C. To compare with the Raman results, we added an offset corresponding to the case temperature (Tcase = 50 °C) to the ΔT measured by thermoreflectance. Using thermoreflectance imaging, we measured the temperature distribution across the region of interest (ROI) on the resistor (Figure 17 right). This allowed us to plot the temperature as a function of the dissipated power, providing critical insights into the thermal behavior of the serpentine resistor under varying electrical loads.

#### 4.1.3. Three-Dimensional Finite Element Simulation Results

**Figure 18:**
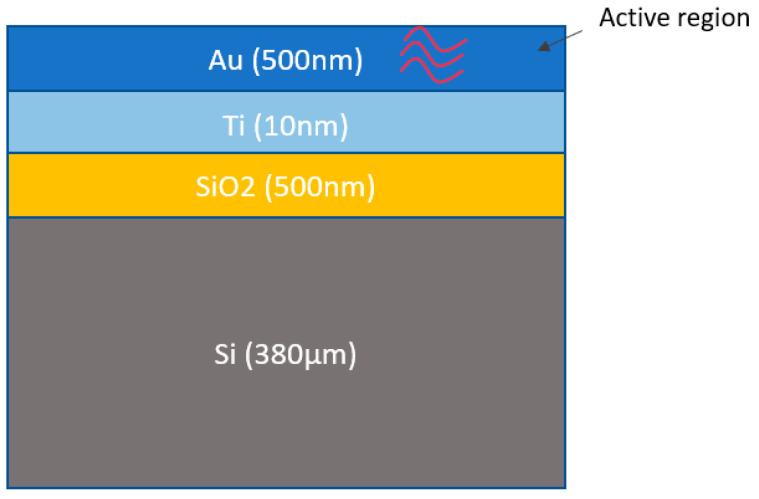
Dimensions of the different layers for the geometry of the 32 × 64 resistor.

In this study, we employed the Finite Element Method (FEM) to model and solve the heat equation for the 32 × 64 resistor, with a geometry that includes several stacked materials with specific dimensions (Figure 18), enabling us to simulate the thermal behavior under varying power dissipation. In our thermal simulation, we adopt a dual approach to modeling thermal conductivity: constant values of thermal conductivity are used for the gold, titanium, and silicon dioxide layers (Table 1), while a temperature-dependent model is implemented for the silicon substrate. Silicon’s thermal conductivity exhibits significant non-linearity with temperature—primarily due to increased phonon scattering at elevated temperatures—which can notably influence the overall thermal gradients and maximum temperatures in the device. The non-linear model of thermal conductivity for the Si is defined as [23]:

(4) $$k\left(T\right)=k_{0}{\left(\frac{T}{T_{0}}\right)}^{-n}$$

As boundary conditions, we applied a bottom surface temperature of 50 °C, and a volumetric internal heat source corresponding to the power levels used during the thermoreflectance measurement phase of our experiments. The volumetric heat source used in the simulation is described as:

(5) $$\dot{q}=\frac{P_{diss}}{2\times V_{Gold\;Serpentine\;Region}}$$

**Figure 19:**
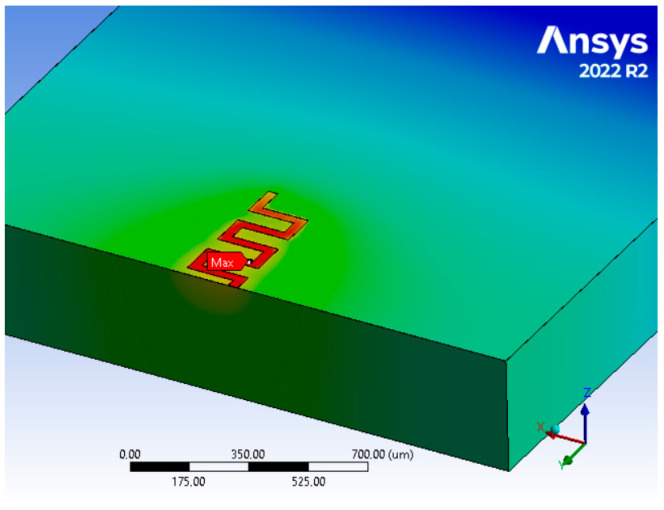
Steady state thermal simulation of the 32 × 64 resistor.

The simulation results (Figure 19) yielded a linear relationship between temperature and dissipated power, closely aligning with the slopes obtained from both the thermoreflectance and Raman shift methods. 

#### 4.1.4. Electrical Method Results

The electrical resistance method operates in two principal steps [19,20]. The first step, known as temperature calibration, involves expressing the device’s resistance *R* as a function of temperature. The relationship obtained can be described by the following linear equation:

(6) $$R=a_{T}\Delta T+b$$

The temperature is provided extrinsically by a thermal chuck on which the sample is placed. The calibration process was conducted by varying the temperature of the thermal chuck from 30 °C up to 125 °C. The second step, known as power measurement, involves expressing *R* as a function of power dissipation, and the relation can be expressed as:

(7) $$R=a_{p}P+b$$

**Figure 20:**
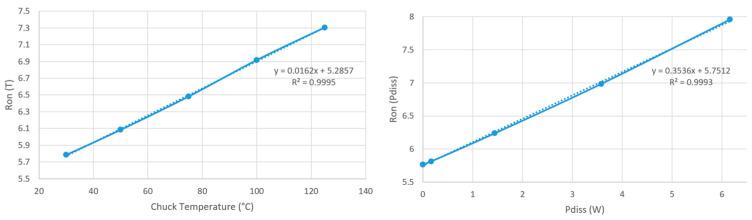
Temperature calibration (left); dissipated power measurements (right).

Following the temperature calibration and power dissipation measurements, we combined the two linear equations to derive the temperature as a function of the dissipated power. Figure 20 shows the results from temperature calibration (left) and the power dissipation measurements (right) on the 32 × 64 resistor.

**Figure 21:**
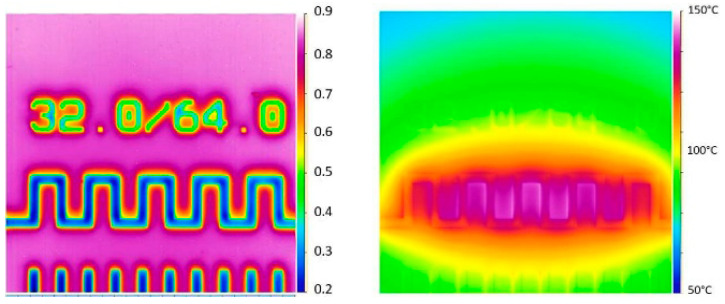
Infrared measurement on 32 × 64 resistor, emissivity of materials (**left**); temperature mapping for 5.7 W dissipated at Tcase = 50 °C (**right**).

**Figure 22:**
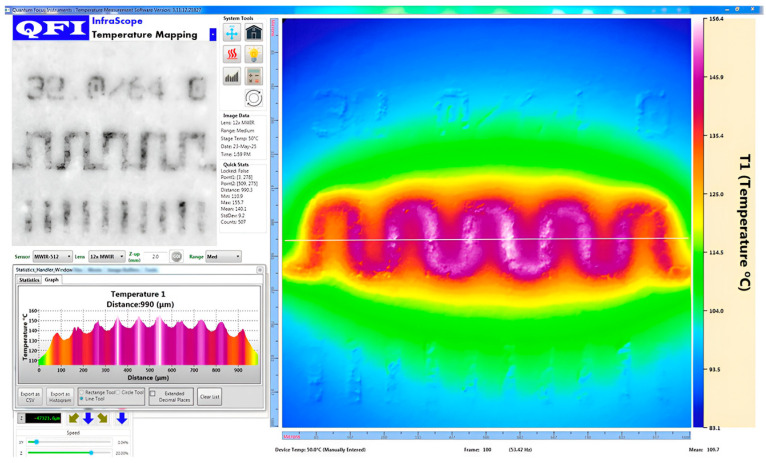
Infrared measurement on 32 × 64 resistor with coating.

Before concluding this measurement campaign, we also performed IR measurements using a QFI (Quantum Focus Instruments) infrared microscope equipped with a 20× objective. The system, named Infrascope, uses an InSb detector operating in the mid-wave infrared (MWIR) band, measuring radiation in the 3–5 μm range. The linear response between the extracted temperature and the dissipated power is confirmed; however, the absolute temperature values are lower than those obtained from Raman thermometry (e.g., around 40 °C at 5.7 W compared to the Eg peak), likely due to low surface emissivity (~0.2) and the lower spatial resolution of the IR technique [2], as shown on the left of Figure 21. The measurement uncertainty in IR thermography strongly depends on material emissivity and can reach errors of more than +20% when the emissivity drops below 0.2. For reference temperature measurements, we used Fluke-calibrated thermocouples compliant with COFRAC (French Accreditation Committee) standards, offering a typical uncertainty of ±0.5 °C. To improve the sensitivity and reliability of the IR measurements, we added a coating layer on top of the resistor surface to enhance emissivity (Figure 22). This approach yielded better results compared to our previous measurements on a similar gold resistor without coating [8].

**Figure 23:**
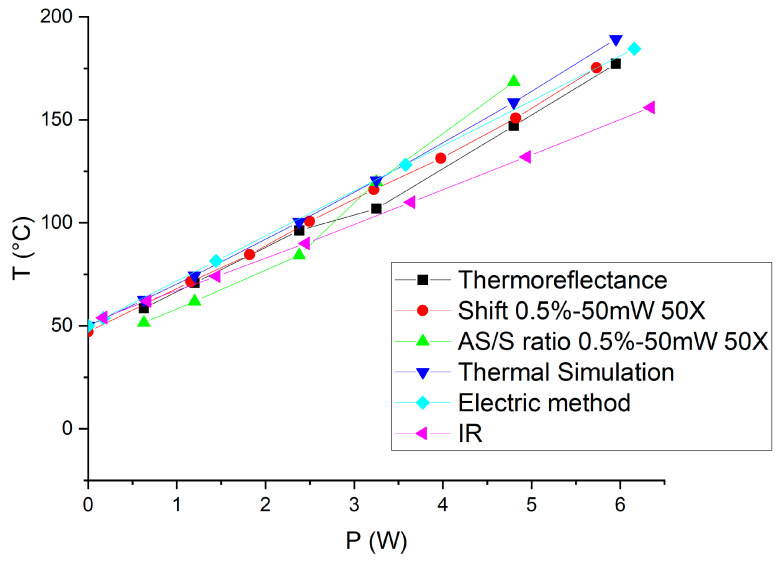
Temperature extraction with the E_g_ Raman peak shift, AS/S E_g_ peak intensity ratio, thermoreflectance, thermal simulation, electrical method, and infrared measurement on the 32 × 64 resistor at T_case_ = 50 °C.

**Figure 24:**
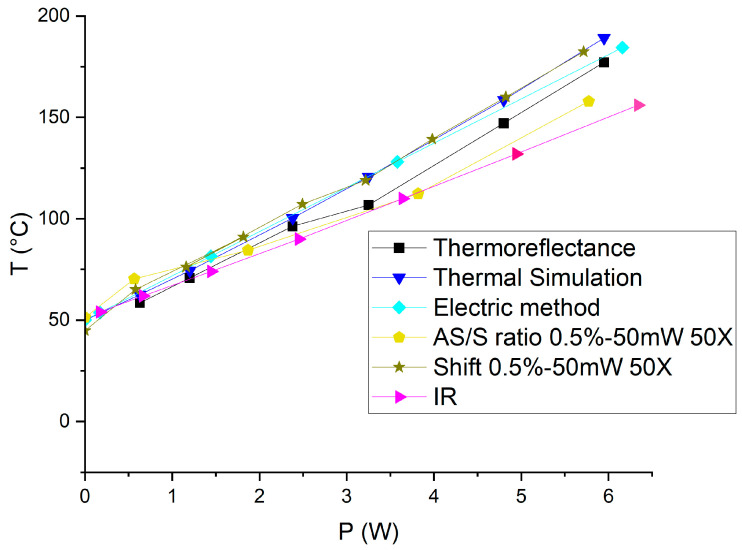
Temperature extraction with the A_1g_ Raman peak shift, AS/S A_1g_ peak intensity ratio, thermoreflectance, thermal simulation, electrical method, and infrared measurement on the 32 × 64 resistor at T_case_ = 50 °C.

The full results are presented in Figure 23 and Figure 24 for this first test vehicle and the estimated thermal resistance (Rth) values obtained from the different techniques, along with their corresponding linear fit parameters, are summarized in Table 2.

It is clear that we obtain very interesting and coherent results which validate the approach of the use of 2D material for measuring temperature with Raman spectroscopy on metals. The thermal simulation, for example, shows a slightly higher slope than Raman or thermoreflectance, likely due to idealized assumptions and uncertainties in the thermal conductivities or boundary conditions, resulting in an overestimation of the thermal resistance. When comparing the shift-based analysis to the AS/S ratio-based method, we observe that the AS/S ratio approaches (for both E_g_ and A_1g_) show lower R^2^ values and higher variability in the extracted parameters. This suggests that peak position tracking (shift) offers a more robust and reliable temperature indicator than intensity ratio methods. The electrical method yields intermediate temperature values, as it reflects a spatial average across the entire resistor whereas the hottest area is at the center of the resistance (Figure 23). However, it results in higher extracted thermal resistance compared to Raman and thermoreflectance measurements. This discrepancy may be attributed to the presence of the physical support holding the gold resistor chip, which introduces additional heat dissipation paths or thermal resistances not present in thermoreflectance measurements performed on an unsupported resistor. Moreover, the higher dissipated power levels used during the electrical measurements may also contribute to the observed difference. In contrast, both thermoreflectance and Raman spectroscopy are highly localized techniques, capturing temperature more precisely near the resistor’s center, where heating is maximal.

### 4.2. SiC JBS Diode

Other experiments were carried out on a power SiC diode. The goal of this analysis is to compare the response for two types of PtSe_2_ patch: transferred patch and grown patch i.e., patterned after the direct synthesis on the diode, and also validate the thermal conductance through the interface between the diode anode metal and the grown 2D material using thermoreflectance.

**Figure 25:**
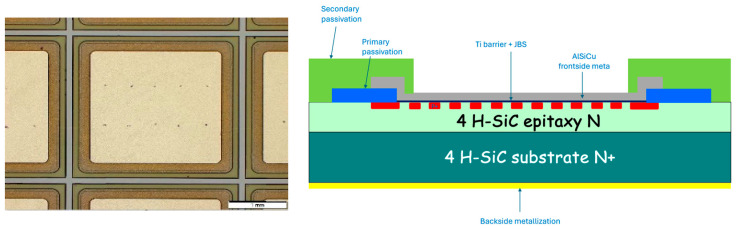
Optical image of an SiC diode (**left**); cross-sectional schematic of the SiC diode (**right**).

The 1.2 kV 20 A power diode manufactured by STMicroelectronics is fabricated on a 180 µm-thick silicon carbide substrate with a Junction Barrier Schottky (JBS) structure. The diode dimension is 3 × 3 mm^2^, the upper anode electrode in Ti/AlSiCu stack is 2.7 × 2.7 mm^2^ (Figure 25). For this study, we used two 20 × 20 mm^2^ wafer cuts with 35 diodes (one with transferred patches, the other with grown patches), each sample was glued with silver paste on a gold-coated AlN base. The analyzed SiC diode, is biased with DC currents (from 0 to 6 A) by using two 500 µm-thick probes for the anode electrode and two wires brazed onto the gold-metallized AlN base for the cathode electrode (Figure 9).

#### 4.2.1. Thermoreflectance Results for the SiC JBS Diode

**Figure 26:**
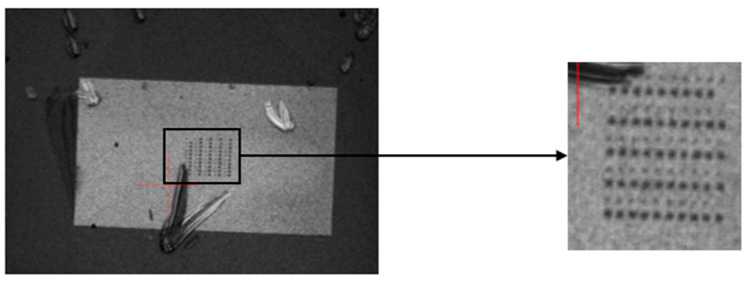
Optical image of the SiC JBS diode with grown 2D material using the thermoreflectance CCD camera.

**Figure 27:**
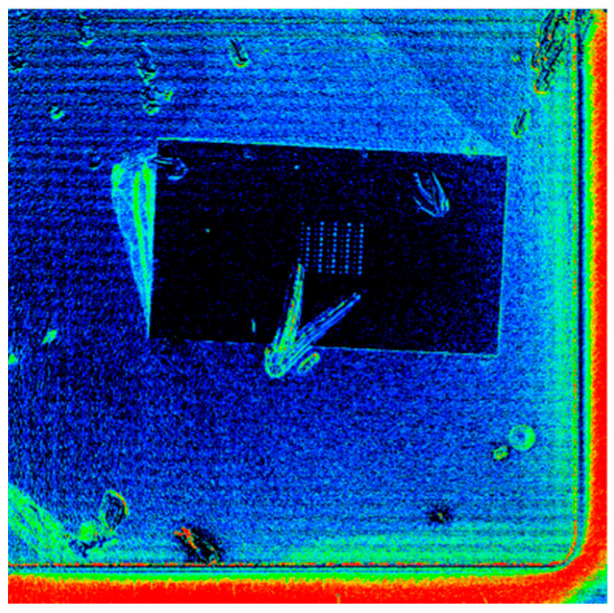
Calibration Image of the SiC JBS diode using a wavelength of 780 nm.

To investigate the thermal response of the SiC diode under electrical load, we performed thermoreflectance measurements using a light source operating at a selected wavelength of 780 nm. Before the measurements, a calibration step was conducted on the grown PtSe_2_ material to determine its thermal coefficient of reflectance as shown in Figure 26. Figure 27 represents the calibration image of the device surface, where each color corresponds to a different extracted thermoreflectance coefficient.The extracted values were $C_{th_{PtSe2}}=3.9\pm 0.7\;E^{-4}K^{-1}$ for the grown 2D material and $C_{th_{AlSiCu}}=1.7\pm 0.2\;E^{-4}K^{-1}$ for the AlSiCu metallization layer.

The experimental procedure was carried out at an initial ambient temperature of 28 °C with a DC current ranging from 0 A to 5 A applied to the diode during the measurement step. Thermoreflectance imaging was performed on both the grown 2D material (PtSe_2_), appearing as dark squares in the optical image, and the AlSiCu metallization, which exhibited a brighter contrast (Figure 26).

**Figure 28:**
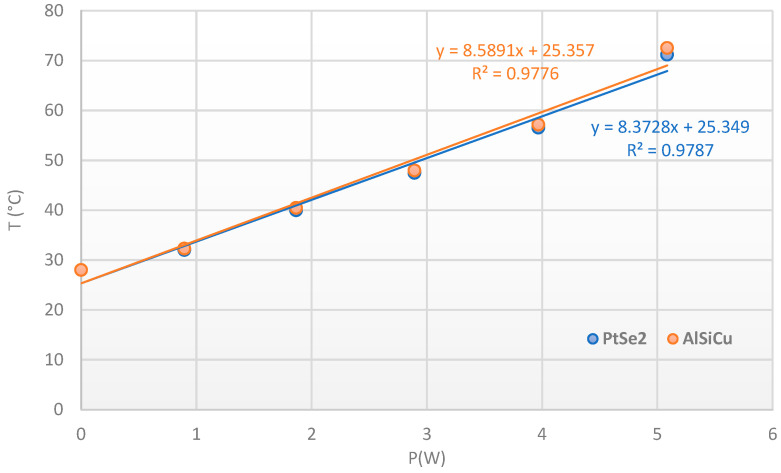
Temperature vs. dissipated power for grown patch and aluminum metallization of the SiC JBS diode.

Interestingly, the temperature evolution observed for both AlSiCu and PtSe_2_ followed the same trend, indicating that both materials experienced an identical thermal response under load (Figure 28). This agreement suggests efficient thermal conductance between the AlSiCu metallization and the grown PtSe_2_ layer.

**Figure 29:**
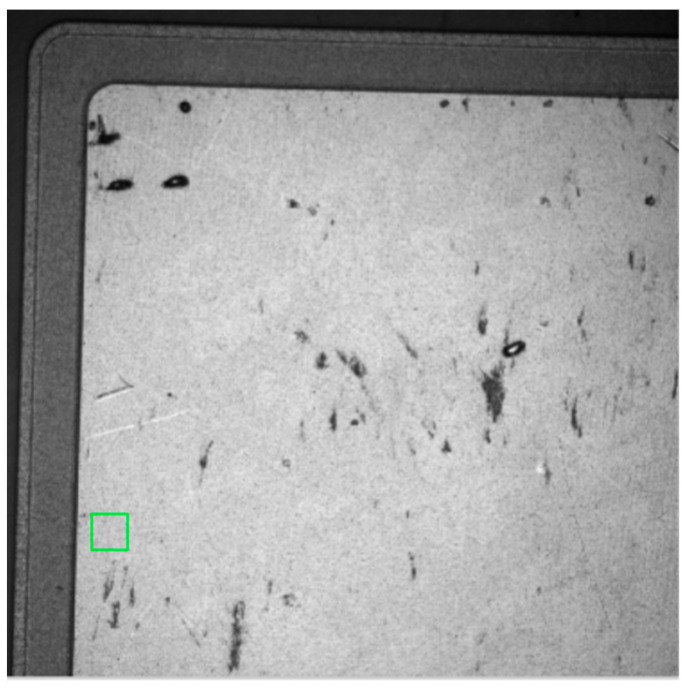
Optical image of an SiC JBS diode without 2D material.

To further investigate the effect of the PtSe_2_ growth process (to pattern the grown patches) on the thermal behavior of the SiC diode, we performed additional thermoreflectance measurements on a similar diode without the 2D material (Figure 29). This comparison aimed to assess whether the growth step alters the diode’s thermal response.

**Figure 30:**
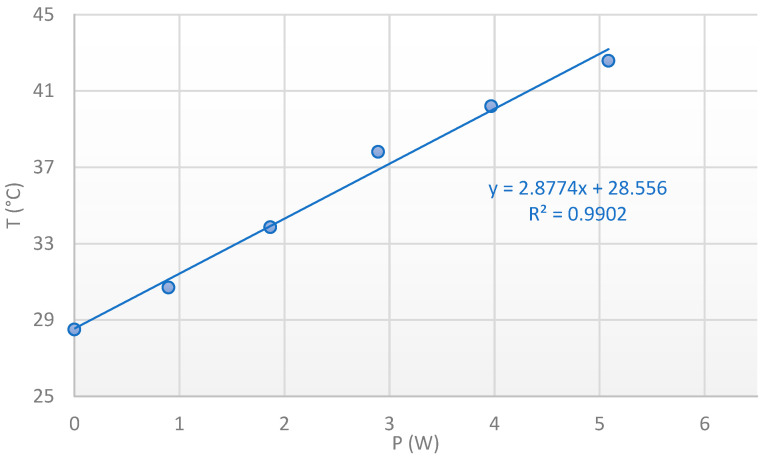
Temperature vs. dissipated power of the SiC JBS diode without 2D material.

Using the same 780 nm wavelength for thermoreflectance imaging, we observed that the thermal response of the unetched SiC diode also follows a linear trend with respect to applied power (Figure 30). However, the extracted slope was 3 times lower than that of the grown PtSe_2_ case. This result indicates that the grown patch process introduces an additional thermal resistance, impacting heat dissipation within the device.

Given that the sample is exposed to a temperature of 400 °C during PtSe2 growth, the associated thermal treatment may modify the interface quality of the frontside or the backside metallization.

#### 4.2.2. Raman Spectroscopy Results for the SiC JBS Diode

According to the results in Section 4.1, we only focused on the Eg peak shift with the temperature, ranging from room temperature to 150 °C for the grown patch. This choice is justified by the better consistency of the E_g_ results compared to those of the A_1g_ peak, and their closer agreement with thermoreflectance and electrical measurements.

**Figure 31:**
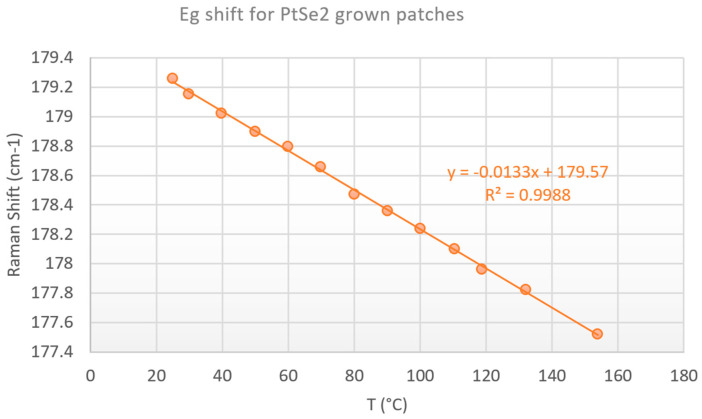
Thermal calibration curve with the Eg Raman peak shift method for grown PtSe_2_ patches on the SiC diode. 50XLF, 3000 lines/mm, edge filter, static regular, 0.25 mW, 30 s, 4 frames.

The calibration curve according to the shift method for the Eg peak is presented in Figure 31 and demonstrates again an excellent linear response with an R^2^ = 0.9988.

**Figure 32:**
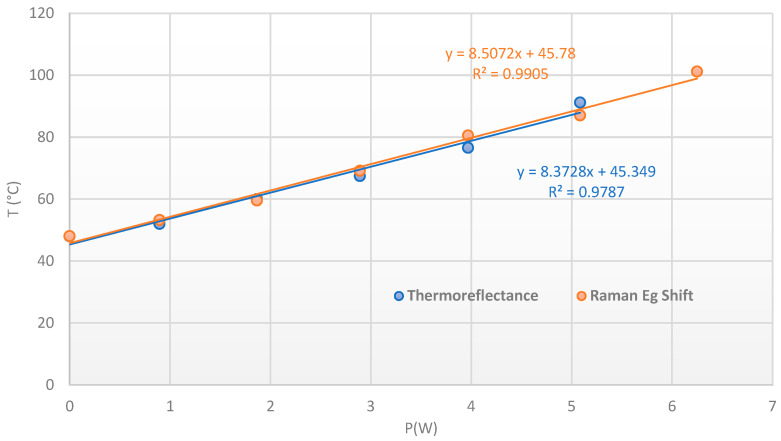
Temperature extraction with the Eg Raman peak shift (orange dots) for grown patch on SiC diode vs. thermoreflectance measurements (blue dots).

To analyze the difference between Raman shift and thermoreflectance results and the increase in the temperature inside the diode, we have to consider Equation (8), where Rth is the thermal resistance (°C/W) and P_DC_ (W) the dissipated power inside the diode:

(8) $$R_{th}P_{DC}=(T_{junction}-T_{case})$$

where to calculate this power, we used the values of the diode voltage given by STMicroelectronics for the different currents. The T_case_ is maintained to 50 °C and to ensure a direct comparison, we added an offset to the temperature values obtained from the thermoreflectance (Figure 32).

**Figure 33:**
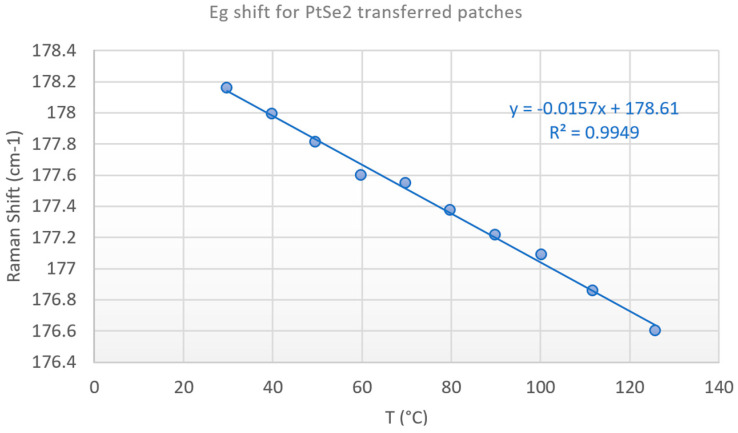
Thermal calibration curve with the Eg Raman peak shift method for transferred PtSe_2_ patches on the SiC diode. 50XLF, 3000 lines/mm, edge filter, static regular, 0.25 mW, 30 s, 4 frames.

To further investigate the thermal behavior of the SiC diode and assess the impact of the PtSe_2_ deposition process, we performed Raman thermometry measurements on a transferred PtSe_2_ layer. The goal was to compare the thermal response of the transferred and grown PtSe_2_ configurations and evaluate potential differences arising from the fabrication process. The Eg peak shift with the temperature ranging from room temperature to 130 °C for transferred patch is presented in Figure 33.

**Figure 34:**
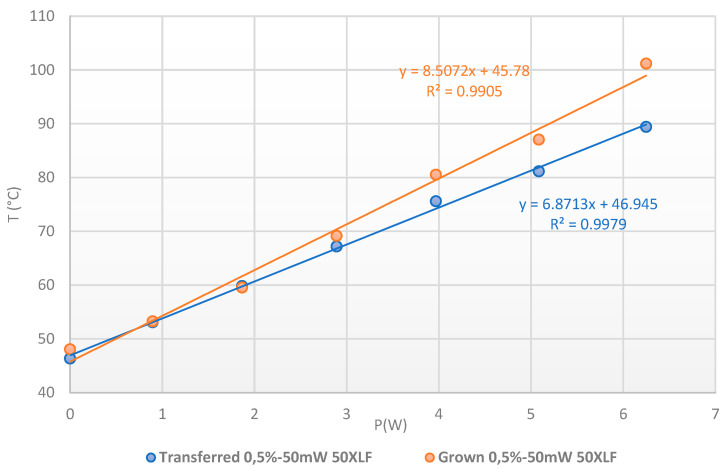
Temperature extraction with the Eg Raman peak shift for transferred patch (blue dots) and etched patch (red dots) on power SiC diode, from 0 to 6 A at Tcase = 50 °C.

The evolution of the temperature as a function of P_DC_ is shown in Figure 34, with an excellent linear fitting and a slope given an Rth of 6.87 °C/W for transferred patch and 8.51 °C/W for grown patch. The difference is directly due to the thermal interface at the SiC/AlN and AlN/Peltier levels, especially for the grown patch (Figure 34). Nevertheless, we can notice that the synthesis at 400 °C does not affect the electrical characteristics of the semiconductor and the upper anode electrode, but it affects the quality and resistivity of the backside metallization. This explains the bad interface with the golded AlN observed in X-ray radiography and the increase in the Rth for the grown patch sample. This means that the growth temperature of PtSe_2_ will have to be reduced to avoid this effect.

**Figure 35:**
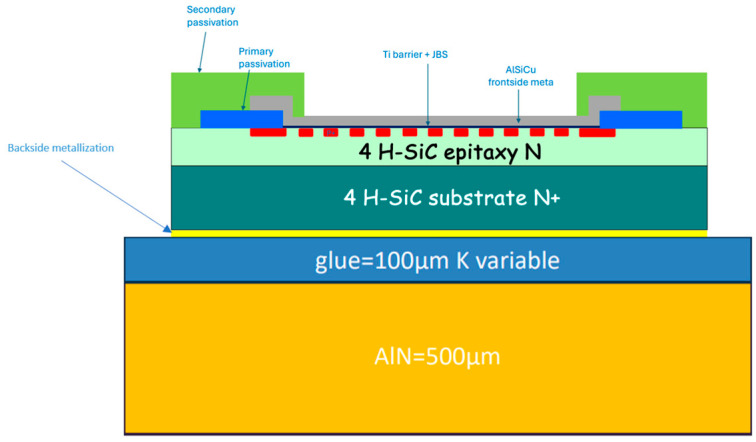
The Schottky-JBS diode mounted on the AlN support.

**Figure 36:**
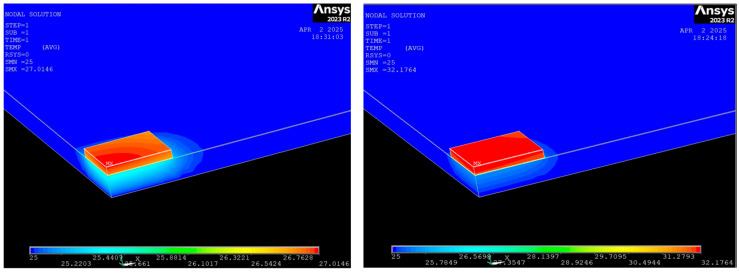
Temperature variation vs. power for thermal paste with k = 2.5 W/mK (**left**) and k = 0.5 W/mK (**right**).

We performed thermal simulation on the SiC JBS diode, specifically focusing on the thermal impact of the silver paste layer. The goal was to understand how variations in the thermal conductivity of this layer influence heat dissipation and overall. The simulated geometry follows the same structure as shown in Figure 25, but now includes two additional layers: the silver paste (15 µm) and the AlN base (500 µm) (Figure 35). These layers play a crucial role in the heat dissipation path, particularly at the interface between the diode and its packaging. For the simulation, we assume a power dissipation of 1 W within the drift region of the SiC diode, and thermal conductivities of the different materials are summarized in Table 3. We then examine two different scenarios by varying the thermal conductivity of the silver paste: a reference one, where the silver paste (EPO-TEK H20E) has the thermal conductivity given by the datasheet, i.e., 2.5 W/mK (Figure 36, left) and a low-conductivity case (0.5 W/mK) (Figure 36, right) representing a more resistive interface due to voids and degradation of the interface.

For the simulation, we assume a double symmetry and we consider only a quarter of the structure. The power of 0.25 W is applied on the surface of the 4H-SiC N layer, corresponding thus to a power of 1 W for the full structure. Moreover, the temperature variation can be directly compared to the slope of the measurement representing T versus power.

**Figure 37:**
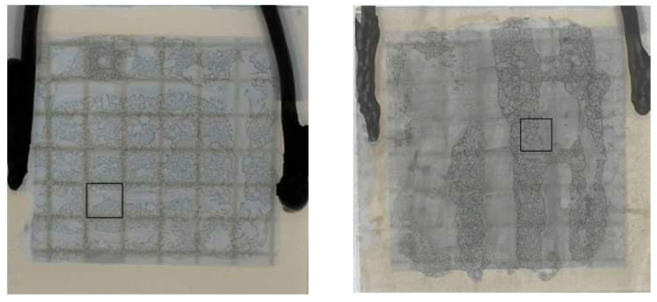
X radiography of the die attach for the two samples; presence of important voids for the etch patch one (**right**).

When the silver paste is unaltered, we obtain almost the same value of 2.14 °C/W as those obtained in Figure 30 (2.87 °C/W). So the simulation is in line with the measurements. Moreover, if we only extract the thermal resistance of the diode alone, we obtain a result close to that given by the ST microelectronics datasheet [26]. When the silver paste is degraded as shown in Figure 37, the behavior can be reproduced by a thermal conductivity of 0.5 W/mK. In that case, it is also in line with the measurements presented in Figure 34.

**Table 3:** Thermal conductivity of materials.

Materials	k (W/m/°C)	
SiC N	390	[27]
SiC N+	300	[27]
Silver paste	2.5/0.5	[28]
AlN	180	
AlSiCu	160	
Backside metallization	40	

## 5. Conclusions

In this work, we have demonstrated the feasibility and effectiveness of using 2D materials for precise thermal mapping of semiconductor-based power devices. By employing Raman thermometry and thermoreflectance measurements, we achieved a comprehensive analysis of temperature distributions at the submicroscale, overcoming the limitations posed by metallization layers that prevent Raman signal acquisition. Our experimental results, obtained through both optical techniques, Raman spectroscopy, and thermoreflectance, exhibited strong congruence with numerical thermal simulations and electrical method. Furthermore, we analyzed the effects of PtSe_2_ growth process, revealing its influence on the thermal resistance of the device. Additionally, we compared two different techniques for integrating 2D materials onto power electronic devices: the transfer technique and the direct growth technique.

Overall, this work validates the use of 2D materials as highly effective thermal sensors, enabling precise temperature measurements on power electronics where conventional Raman thermometry is not applicable. These findings pave the way for further optimization of thermal characterization techniques and for the integration of 2D materials into advanced thermal management strategies.

## Figures and Tables

**Table 1:** Thermal conductivity table.

Materials	k (W/m/°C)	n	
Gold (Au)	315	-	
Titanium (Ti)	17	-	[24]
Silicon Dioxide (SiO_2_)	1.28	-	[25]
Silicon (Si)	145	1.3	

**Table 2:** Summary of linear fit parameters and thermal resistance estimates from multiple techniques applied to the 32 × 64 resistor.

Method	Symbol	Slope (Rth)	Intercept (T_0_)	R-Square
Thermoreflectance	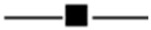	21.898	42.996	0.9925
Raman Eg shift	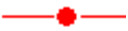	22.099	45.501	0.9972
Raman Eg AS/S ratio	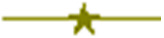	19.884	49.256	0.9937
Raman A_1g_ shift	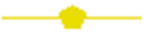	23.204	48.139	0.9972
Raman A_1g_ AS/S ratio	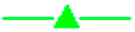	17.209	53.915	0.9814
Electric method	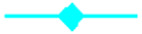	21.827	50	1
Thermal simulation	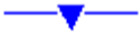	23.343	47.251	0.9981
Infrared method	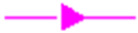	16.509	50.488	0.9996

## Data Availability

Data supporting the findings of this study are available from the corresponding author upon reasonable request. No publicly archived datasets were generated during the current study.
